# The Impact of COVID-19 Restrictions on Psychological Distress in Family Caregivers of Children with Neurodevelopmental Disability in the UK

**DOI:** 10.1007/s10803-021-05132-3

**Published:** 2021-07-20

**Authors:** Karri Gillespie-Smith, Doug McConachie, Carrie Ballantyne, Bonnie Auyeung, Karen Goodall

**Affiliations:** 1 Department of Health and Clinical Psychology, School of Health in Social Science, University of Edinburgh, Edinburgh, Scotland, UK EH8 9AG; 2 Division of Psychology, University of West of Scotland, Paisley, Scotland, UK PA1 2BE; 3 Department of Psychology, School of Philosophy, Psychology and Language Sciences, University of Edinburgh, Edinburgh, Scotland, UK EH8 9AG

**Keywords:** COVID-19, Caregivers, Coping, Challenging behaviours, Neurodevelopmental disabilities, Psychological distress

## Abstract

Caregivers of a child with a neurodevelopmental disability are more vulnerable to mental health difficulties. These difficulties are influenced by the child’s challenging behaviours, and the caregiver’s coping strategies; factors impacted by the COVID-19 pandemic. An online mixed methods survey was conducted on caregivers of children with neurodevelopmental disabilities (*n* = 43) and children who are typically developing (*n* = 67). The results showed that presence of challenging behaviours related to neurodevelopmental disability, and caregiver coping strategies predicted caregiver psychological distress during lockdown. Themes that emerged included ‘confusing messages and guidance’, ‘loss of freedom’ and ‘unsupported and forgotten’. The results demonstrate the pressing need for the implementation of appropriate support to protect the mental health of caregivers across the UK.

COVID-19 was first discovered in December 2019 in Wuhan China, and with an aggressive rate of infection, the World Health Organisation (WHO) declared the virus to be a ‘Public Health Emergency of International Concern’ in January 2020. The first UK-wide lockdown was implemented on 23rd March 2020, which established Public Health Safety Measures (PHSM) to reduce the spread of the virus (Cabinet Office 2020). These UK-wide PHSM were in place when recruiting began for the current study as this was a crucial time to explore mental health in vulnerable groups. The current project aims to investigate how the UK COVID-19 response impacted children’s challenging behaviours, and to examine psychological distress and coping strategies in caregivers of children with and without neurodevelopmental disabilities.

Caregivers of a child with a neurodevelopmental disability are more vulnerable to mental health difficulties when compared to carers of typically developing children (Eisenhower et al., 2005; Gallagher, et al., 2008; Herring et al., 2006). Social support and the severity of the disability, psychiatric disorder and children’s challenging behaviours are all key factors that contribute to the psychological distress observed in caregivers (e.g. Blacher & McIntyre, 2006; Unwin & Deb, 2011; Weiss, 2002; White & Hastings, 2004). Neurodevelopmental disabilities are characterised by social, cognitive, and adaptive skill deficits (Zayac & Johnston, 2008), which can be associated with challenging behaviours (Lee, et al., 2008), including verbal and/or physical aggression, self-injury, disturbed sleep, over- or inactivity, sexual or socially inappropriate behaviours and destructive tendencies (Benson, & Brooks, 2008; Myrbakk & Von Tetzchner, 2008). Management of challenging behaviours impacts both the individuals’ quality of life and opportunities (e.g. career, social, etc.) but also poses a significant additional burden on caregivers, leading to increased stress levels (Blacher & McIntyre, 2006; McConnell & Savage, 2015).

Challenging behaviours indicate levels of severe mental stress and distress reactions in those with intellectual disabilities (Courtney & Perera, 2020). These behaviours are exacerbated by disruption to day-to-day routines (adaptive functioning), or restrictions on enjoyed activities (Borthwick-Duffy, 1994; NICE guidelines – NG11; published May, 2015) and are conditional on the level of disability (Davies & Oliver, 2016), depression (Davies & Oliver, 2014) and anxiety (Koritsas, & Iacono, 2015; Rzepecka, et al., 2011). It could be hypothesised that the COVID-19 pandemic and related limitations to routine and lockdown arrangements (Anderson et al., 2020) are likely to trigger or exacerbate these factors. Conversely, it could be hypothesised that for some children, increased consistency associated with being at home, reduction of school pressures and bullying may reduce anxiety, actually leading to a reduction in challenging behaviours (Cappadocia et al., 2012; Weiss et al., 2015). It is therefore important to examine how lockdown restrictions to education, respite and support services (that are known to reduce caregiver stress; for a review see Chan, & Sigafoos, 2001) will impact both the carer coping strategies and children’s challenging behaviours. Indeed, there is evidence showing an increase in requests for psychotropic medication across Intellectual Disability (ID) services to manage children’s challenging behaviour during the pandemic (BMJ 2020; 369: m1609). This may indicate changes in the child behaviours or caregivers’ perceptions of coping. It is therefore, crucial to understand the impact of the COVID-19 restrictions on both behaviours that challenge in children and caregivers’ coping strategies.

Children’s challenging behaviours are positively associated with parental levels of depression and anxiety (Baker et al., 2003; Blacher & McIntyre, 2006; Floyd & Gallagher, 1997), with challenging behaviours and number of co-occurring conditions adding to perceived stress in caregivers (Snowdon, et al., 1994). Challenging behaviours are not the only factor that influences caregiver distress. The wider caregiver context suggests; caregiver sleep quality, excessive caregiving demands, lack of child responsiveness and perceived caregiver burden are all predictors of caregiver psychological distress (Brummett et al., 2006; Snowdon, et al., 1994; Thompson, et al., 2008). Sleep quality (crucial in health and wellbeing) is related to psychological problems in caregivers (Brummett et al., 2006; McCurry, et al., 2007). Perceived caregiver burden is another source of psychological distress in caregiver groups (Clyburn, et al., 2000; Gallagher, et al., 2008; Maes et al., 2003). Perceived burden includes overload, embarrassment, guilt, resentment, isolation, feelings of entrapment and loss of control (Zarit, et al., 1980), with the guilt component being the greatest predictor of depression and anxiety in caregivers of children with intellectual disabilities (Gallagher et al., 2008). Additionally, due to higher caregiver burden, parents who have children with intellectual disabilities experience more limited employment opportunities, which is proposed to further compound feelings of isolation and low self-esteem (Shearn & Todd, 2000). COVID-19 has been reported to have significantly increased the perceived levels of strain and burden in caregivers who have children with Special Educational Needs and Disabilities (SEND) (Dhiman et al., 2020).

Caregivers have often identified respite services as the most helpful service received (Sherman, 1988) and has been shown to prevent or lessen caregiver stress and burnout (Sherman, 1995). Respite is also important for the whole family unit since it has a positive impact on emotional wellbeing and physical strains (Joyce, et al., 1983; Wilkie & Barr, 2008). Reduction in clinical, educational and respite services for children with neurodevelopmental disorders during COVID-19 presents significant challenges to caregivers (Neece et al., 2020; O’Hagan & Kingdom, 2020).

In addition to respite services, social support specifically has been reported to mitigate the distress reported in parents (Dunn et al., 2001) with research showing that parents who have high levels of social support also show better psychological adjustment (Duis et al., 1997; Dunn et al., 2001; Gray & Holden, 1992). This social support may simply come in the form of societal understanding of a child’s condition (Hughes et al., 1993). Greater distress is reported in those families who perceive greater caregiver burden and limited social support (Bailey et al., 1994; Dunn et al., 2001; Gallagher et al., 2008).

Previous research has suggested that disruption to routines and restrictions on social activity not only impact how caregivers perceive their situation but also the coping strategies they employ (Cramm, & Nieboer, 2011). Recent research on the impact of COVID-19 on caregivers of children with neurodevelopmental disorders has highlighted that caregivers need to spend more time on self-care that focuses on social support and social opportunities and not just traditional areas (i.e. exercise, stress management, smoking cessation, etc.) to improve mental health (Chafouleas et al., 2020). To date, research has reported a variety of specific coping strategies that positively impact mental health outcomes in carers of children with neurodevelopmental disabilities. These coping strategies include cognitive reframing and acceptance (Hastings, et al., 2002; O Donnchadha, 2018); positive re-interpretation (Smith, et al., 2008) and active emotional coping strategies (Ganjiwale, et al., 2016).

Furthermore, adaptation to a child’s challenging behaviours requires parents to regulate their own behaviour in relation to their own interpretation and reaction to their child’s behaviour. Where caregivers experience difficulty in managing their own emotional responses, they have been shown to be more likely to respond with harsh discipline or overt expressions of despair, contempt or depression (Bugental & Happaney, 2004). Given the additional external stressors that many carers will experience in the current times (for example, financial, employment or other family concerns), recognition of the bi-directional relationship between caregiver coping strategies and challenging behaviours is necessary. A recent review highlighted how the complex interplay of factors including challenging behaviours, caregiver stress, coping strategies, and social support contribute to mental health outcomes in caregiver groups (for a review see Isa et al., 2016). How these important interdependent factors have been impacted by COVID-19 needs to be investigated since recent research suggests that these issues have been further compounded due to the PHSM (Dhiman et al., 2020; Neece et al., 2020; O’Hagan & Kingdom, 2020). In addition, research to date has examined the impact of COVID-19 on caregivers of children with ID focusing independently on either coping strategies (e.g. Willner et al., 2020) or levels of children’s challenging behaviours (e,g, Bailey et al., 2021; Mutluer et al., 2020). The current study examines both of these interdependent factors within the same UK sample, providing novel insight into how both parental coping strategies and children’s challenging behaviours impact parental mental health during COVID-19 restrictions.

The first aim of this project is (1) to investigate the relationships between children’s challenging behaviours, coping strategies and psychological distress during COVID-19 Lockdown in caregivers of children with neurodevelopmental disabilities. Currently, little is known about how COVID-19 restrictions have impacted caregivers’ experiences and perceptions of the pandemic restrictions. Therefore the current study also aimed to survey caregivers who have children with neurodevelopmental disabilities across the UK to determine: (2) Their experiences of the pandemic and restrictions; (3) Their awareness of how the pandemic has influenced their child’s behaviours (4) How satisfied they are in their dealings with support services; and (5) their own needs as caregivers.

## Method

### Participants

Caregivers of typically developing children (*n* = 98) and children with a neurodevelopmental disability (*n* = 46) were initially recruited using opportunistic sampling during the period of restrictive Lockdown in the UK (between April and June 2020) as part of an ongoing survey. Both groups were recruited via online adverts being posted on relevant social media groups and websites (e.g. parenting group webpages, parenting charities, neurodevelopmental disability webpages, neurodevelopmental charities etc.). Due to the opportunistic sampling the groups were not matched based on levels of distress before the COVID pandemic. A proportion of caregivers of typically developing children did not continue the survey beyond the demographic questionnaire and were omitted. Thus, 67 participants who identified themselves as caregivers of typically developing children were retained. Caregivers of children with a neurodevelopmental disability were asked to self-report recorded diagnoses received from clinical and/or pediatric services. Three participants were removed because their children had no diagnosis of neurodevelopmental disorder. The remaining 43 participants reported their child as having diagnoses of Autism Spectrum Condition (ASC; *n* = 27); ASC and Intellectual Disability (*n* = 1); Intellectual Disability (ID; *n* = 6); Attention Deficit Hyperactivity Disorder (ADHD; *n* = 5); Developmental Coordination Disorder (DCD; *n* = 1) and Dyspraxia (*n* = 1); Chromosome 7Q deletion (*n* = 1); and cerebral palsy (*n* = 1). For more information regarding participant characteristics, please see Table 1. A Gpower calculation based on running regression models with 8 predictors (controlling for variables gender and age of child), with a moderate effect size of 0.3 indicated that a sample of *n* = 107 was required. Therefore the final sample size (*n* = 110) was still within these parameters.

**Table 1:** Demographic characteristics

	Caregivers of children with neurodevelopmental disorders		Caregivers of children who are typically developing	
	*n*	%	*n*	%
Total	43	100	67	100
Caregiver relationship				
Parent	40	94	65	97
Sibling	1	2	2	3
Foster carer	1	2	–	–
Grandparent	1	2	–	–
Caregiver Qualification				
Postgraduate	9	21	16	24
Undergraduate	14	33	25	37
Vocational training	9	21	15	22
Highers/diplomas	10	23	10	15
None	1	2	1	2
Marital status				
Married	25	58	43	64
Partner	9	21	13	20
Single	8	19	9	13
Other	1	2	2	3
Child characteristics				
Gender male	33	77	39	58
Female	10	23	28	42
Age (mean in years)	11.2		8.1	
Other dependents	–	–		
None	11	25	14	21
One child	16	37	37	55
Two children	13	31	13	19
Three children	3	7	1	2
Four or more children	–	–	2	3

### Procedure

Following ethical approval granted from the University of Edinburgh Ethics Committee, participants were recruited online (via charity websites, social networking sites and parent group pages) to take part in the online survey which took approximately 45–50 min to complete. The surveys were presented via the Qualtrics survey system. This system first presented the participant info screen, followed by the consent screen so consent was obtained before any questionnaires were presented.

### Measures

#### Survey on COVID-19

A questionnaire was developed and consisted of 40 items asking basic demographic data (age of the child, marital status, employment status, etc.) and asking categorical questions about the impact of COVID-19 and how the government restrictions had impacted their family situation, living arrangements, and services/respite support. Finally, participants were invited to respond to open text boxes about their experiences of the pandemic, and related restrictions, how they impacted their child’s behaviours, experiences of support services at the time, and opinions of what support caregivers needed during COVID-19 restrictions. The survey took approximately 15 min to complete. Caregivers were then asked to complete the following psychometric scales during the COVID-19 lockdown restrictions.

#### Anxiety and Stress of Parents/Caregivers

The Depression, Anxiety and Stress Scale (DASS-21; Lovibond & Lovibond 1995), a self-report questionnaire consisting of 21 items, with 7 items per subscale: depression, anxiety and stress. Participants are asked to score every item on a 3-point likert scale from 0 (*did not apply to me at all*) to 3 (*applied to me very much*). Numerous studies have reported favourable psychometric properties of the DASS in adults with anxiety and/or mood disorders in both community and clinical samples (Antony et al., 1998; Clara, et al., 2001). DASS total scores were used as a measure of parental psychological distress with higher scores indicating higher levels of anxiety and depression (Beaufort, et al., 2017). Studies have demonstrated excellent internal consistency of the DASS scales in both the 42- and 21-item (DASS-21) versions: ranging from 0.81 to 0.97 (Gloster, et al., 2008).

#### Child Challenging Behaviour

The Developmental Behaviour Checklist—P24 (DBC – P24; Taffe, et al., 2007) is a 24-item checklist which is a shorter form of the DBC which is a 96 item scale (Einfeld & Tonge 1994, 1995) specifically designed to assess behavioural and emotional disturbance in children/adolescents with intellectual disability. The instrument has 24 items (e.g. impatience, lack of affection, over-excitement and repetition) that are scored based on a three-point likert scale from 0 (not true) to 2 (very true). The DBC-P24 performs very well in terms of low bias and high precision in cross-validation and displays excellent sensitivity and specificity properties (Taffe, et al., 2007). A total behavior problem score was calculated by summing the scores with higher scores indicating higher levels of challenging behaviours (as perceived by caregivers).

#### Caregiver Coping Strategies

The Brief COPE (Carver, 1997) a shortened version of the COPE, asks 28 questions on a four-point Likert scale, ranging from 1 (*I haven’t been doing this at all*) to 4 (*I’ve been doing this a lot*), where two items each form 14 sub-scales with each subscale showing good internal consistency reliability indicated by the Cronbach’s alpha values ranged from 0.50 to 0.90. These subscales include active coping, planning, positive reframing, acceptance, humour, turning to religion, using emotional support, using instrumental support, self-distraction, denial, venting, substance use, behavioural disengagement, and self-blame. Active coping is the process of taking active steps to try to eliminate the stressor or to reorganise its effects. Planning; consists of thinking about how to handle a stressor which engages with action strategies, i.e. how best to cope with the problem. Positive reframing is a type of emotion-focused coping which involves construing a stressful transaction in positive terms and may lead a person to show problem-focused coping actions. Self-distraction refers to focusing on alternative activities. Denial is defined as trying to act like the stressor is not real or refusing to believe that the stressor exists. The opposite of denial is acceptance, which is a functional coping reaction in which an individual acknowledges the reality of a stressful situation in an effort to deal with the situation (Carver, 1997; Carver et al., 1989).

Use of emotional support is a type of emotion-focused coping which consists of obtaining understanding, sympathy and/or moral support from others. Instrumental support is an aspect of problem-focused coping, which involves seeking out information, help and/or advice (Carver et al., 1989). Behavioural disengagement is a form of helplessness or giving up on attaining goals to solve the problems. Venting is the tendency to ventilate feelings about whatever distress or upset individual is experiencing. Religion is also an active coping tactic in which the individual tends to turn to religion. Humour involves making jokes or fun of a stressful situation. Self-blame refers to criticising oneself for responsibility during the situation. Substance use coping means taking alcohol or other drugs to deal with stressors (Carver, 1997).

This scale has been used widely in groups of caregivers of children with IDs and developmental disorders (e.g. Benson, 2010; Ganjiwale et al., 2016; Hastings et al., 2005; Isa et al., 2017; Panicker and Ramesh, 2019). The scale provided information on each parent’s abilities to cope, perceived social support and self-regulation. Higher scores reflect a higher tendency to implement the specified coping strategies.

### Data Analysis

Normality was assessed using the Shapiro–Wilk test which showed that for the caregivers of children with neurodevelopmental disabilities several of the variables from the Brief COPE significantly departed from normality (all subscales of the Brief COPE were ps < 0.05 except Self Distraction and Planning). For caregivers of typically developing children, all variables (Psychological Distress, Challenging Behaviours and COPE subscales) showed non-normal distributions. Visual inspections of histograms and Q-plots showed that the data were positively skewed; therefore, square root transformations were applied before Pearson correlations were carried out separately for each group (caregivers of children with neurodevelopmental disorders and children who are typically developing). Bonferroni corrections for multiple comparisons were also applied throughout. Based on significant zero-order correlations, multiple linear regressions analyses were conducted to determine independent predictors of parental distress.

In addition to the surveys, participants were also asked to identify and explain key difficulties during the COVID-19 pandemic. The data was analysed using thematic analysis (Braun & Clarke, 2006). The analysis was approached from a qualitative philosophical paradigm with emphasis placed on an individual experience and meaning being contextual, with multiple realities, and with researcher subjectivity acknowledged (Braun & Clarke, 2006; Braun et al., 2019). The process involved six phases as outlined as being consistent with high quality reflexive thematic analysis (Braun et al., 2019): (1) familiarisation with the data; (2) coding of data in sections relating to research questions; (3) initial themes and unifying patterns examined; (4) potential themes reviewed, developed and cross-checked with source data; (5) a further alteration and review of possible themes relative to research aim; (6) the final themes generated. Phases 1 to 3 were conducted by one researcher, and 4 to 6 were conducted by the same researcher and reviewed by a second researcher. The aim was to identify items at both the semantic and latent level to ensure quality and credibility in the process (Terry et al, 2017). Data saturation, as defined by Fusch and Ness (2015), may not have been achieved as it cannot be ruled out that new themes would have emerged with more responses. Appropriate quotes were incorporated into the results section to highlight ideas under discussion.

## Results

### Correlation Effects

Pearson correlations coefficients determined that there were several significant relationships between child challenging behaviours, caregiver coping scores and psychological distress for each group. Age and sex were not significantly associated with any variables. There were several moderate to large associations between variables in both groups. In the caregiver group who have children with neurodevelopmental disabilities the child’s challenging behaviours were significantly related to caregiver psychological distress (*r* = 0.543, *p* < 0.003). In addition, coping strategies, were also related to psychological distress, such as denial (*r* = 0.528, *p* < 0.003) and behaviour disengagement (*r* = 0.462, *p* < 0.003). In the group of caregivers whose children were typically developing, significant relationships were observed between coping strategies such as substance Use (*r* = 0.628, *p* < 0.003), Instrumental use (*r* = 0.455, *p* < 0.003), behaviour disengagement (*r* = 0.608, *p* < 0.003), humour (r = 0.468, *p* < 0.003) and psychological distress (please see correlation results in full in Tables 2 and 3).

**Table 2:** Pearson’s correlations between days schools were closed, caregiver psychological distress, coping strategies and children’s challenging behaviours in caregivers of children with neurodevelopmental disabilities

	2	3	4	5	6	7	8	9	10	11	12	13	14	15	16	17: Self blame
1. Days schools were closed	.454	.476^*^	.344	−.061	.274	.330	.466	.525^**^	.606^**^	.359	−.169	−.199	−.359	.046	.302	.434
2. Caregiver distress	_	.543^**^	.280	.032	.528^**^	.051	−.110	−.028	.462^**^	.269	−.344	−.015	−.325	−.230	−.136	.321
3. Child’s challenging behaviours		_	.022	.075	.143	.205	.156	.334	.289	.404	.080	.219	−.233	.243	.165	.418^*^
4. COPE: self distraction			_	.289	.380	.179	.351	.334	.112	.384	.182	.257	.141	.197	.224	.480^**^
5. COPE: active coping				_	.364	.122	.493^**^	.378	−.108	.515^**^	.260	.467^**^	.078	.010	−.114	.189
6. COPE: denial					_	.136	.021	.141	.146	.403^*^	.165	.219	−.191	−.031	−.138	.411^*^
7. COPE: substance use						_	.384	.202	.241	.137	.049	.144	.137	.354	.135	.492^**^
8. COPE: use of emotional support							_	.744^**^	.086	.532^**^	.303	.243	.246	.288	.392	.216
9. COPE: use of instrumental support								_	.279	.677^**^	.593^**^	.425^*^	.341	.575^**^	.557^**^	.462^**^
10. COPE: behavioural disengagement									_	.182	−.230	−.123	−.213	.021	.025	.441^*^
11. COPE: venting										_	.378	.521^**^	.241	.292	.393	.481^**^
12. COPE: positive reframing											_	.525^**^	.506^**^	.758^**^	.561^**^	.320
13.COPE: planning												_	.199	.438^*^	.481^**^	.298
14. COPE: humour													_	.513^**^	.500^**^	−.012
15. COPE: acceptance														_	.669^**^	.431^*^
16. COPE: religion															_	.190

^*^Correlation is significant to .01. ^**^Correlation is significant to .003 (following Bonferroni Correction; two tailed)

**Table 3:** Pearson’s correlations between days schools were closed, caregiver psychological distress, coping strategies and children’s challenging behaviours in caregivers of children who are typically developing

	2	3	4	5	6	7	8	9	10	11	12	13	14	15	16	17:self blame
1. Days schools were closed	.132	−.271	−.485^**^	−.176	−.080	.332	−.020	.331	.208	.048	.080	−.122	.022	−.292	−.042	.125
2. Caregiver distress	_	.242	−.142	.039	.108	.628^**^	.155	.455^**^	.608^**^	.275	.121	.291	.468^**^	−.270	−.028	.405^*^
3. Child’s Challenging behaviours		_	.290	.310	.332	−.107	.260	.229	.201	.203	.143	.197	.146	.161	.052	.107
4. COPE: Self distraction			_	.362^*^	.076	−.356^*^	.083	−.105	−.169	.139	.465^**^	.339	.237	.597^**^	.311	.112
5. COPE: active coping				_	.033	−.114	.198	.295	−.057	.045	.180	.523^**^	−.217	.154	.048	−.088
6. COPE: denial					_	.016	.226	.193	.239	.140	−.009	−.139	.287	−.245	−.019	.244
7. COPE: substance use						_	.057	.274	.477^**^	.051	−.103	.005	.200	−.394^*^	−.236	.204
8. COPE: use of emotional support							_	.515^**^	.217	.498^**^	.174	.470^**^	.243	.017	.330	.622^**^
9. COPE: use of instrumental support								_	.476^**^	.440^**^	.216	.467^**^	.113	−.246	.172	.375^*^
10. COPE: behavioural disengagement									_	.293	.257	.274	.076	−.225	−.082	.495^**^
11. COPE: venting										_	.400^**^	.460^**^	.258	.030	.397^**^	.471^**^
12. COPE: positive reframing											_	.476^**^	.208	.559^**^	.307	.345^*^
13. COPE: planning												_	.109	.218	.375^*^	.340
14. COPE: humour													_	.130	.273	.369^*^
15. COPE: acceptance														_	.230	−.035
16. COPE: religion															_	.256

^*^Correlation is significant to .01. ^**^Correlation is significant to .003 (following Bonferroni Correction; two tailed)

### Regression Analysis

A series of multiple regression analyses was conducted, to determine the independent effect of group (ID or TD), level of child challenging behaviours and specific coping strategies on caregiver psychological distress. In each model, group was entered as a dichotomous variable at step 1, challenging behaviours at step 2 and coping strategy at step 3. Coping strategies were selected if there were significant zero order correlations with caregiver distress. Multiple analyses were conducted to avoid multicollinearity with coping strategies,

Tables 4, 5 and 6 displays the results of the first sequential multiple regression (Model 1 with Group as the only predictor explained 71.1% of variance and was significant *F*(1,93) = 232.71, *p* = 0.000. Model 2 where Challenging Behaviour Score was added explained significantly more variance (*R*^*2*^ change = 0.084, *F*(1,92) = 38.093, *p* = 0.000). The model explains 79.4% of the variance in psychological distress in caregivers (Adjusted R^2^ = 0.794). Model 3, in which Denial Coping strategy was added explained yet more variance and this increase was also significant (*R*^*2*^ change = 0.009, *F* (1,91) = 4.242, *p* = 0.042). Model 3 explains 80.1% of the variance in psychological distress in caregivers (Adjusted *R*^*2*^ = 0.801) and was significant *F*(3,91) = 126.892, *p* = 0.000.

**Table 4:** Unstandardized and standardised regression coefficients for the variables entered in to the model examining the role of Group, Challenging Behaviours (DBC Score) and Denial Coping Strategy as control variables with outcome variable being psychological distress

Variable	B	SE B	β	*P*	95% CI
Intercept	−6.653	3.037		.031	[−12.685, −.621]
Group	16.441	2.164	.541	.000	[12.144, 20.739]
DBC score	1.173	.189	.434	.000	[.797, 1.549]
Coping denial	3.311	1.608	.098	.042	[.118, 6.505]

**Table 5:** Unstandardized and standardised regression coefficients for the variables entered in to the model examining the role of Group, Challenging Behaviours (DBC Score) and Substance Abuse Coping Strategy as control variables with outcome variable being psychological distress

Variable	B	SE B	Β	*p*	95% CI
Intercept	−.604	3.606		.867	[−7.767, 6.558]
Group	15.582	2.343	.513	.000	[10.928, 20.236]
dbc score	1.189	.195	.440	.000	[.801, 1.577]
Coping Substance abuse	−.067	1.651	−.002	.968	[−3.347, 3.213]

**Table 6:** Unstandardized and standardised regression coefficients for the variables entered in to the model examining the role of Group, Challenging Behaviours (DBC Score) and Behaviour Disengagement Coping Strategy as control variables with outcome variable being psychological distress

Variable	B	SE B	β	*p*	95% CI
Intercept	−10.847	4.194		.011	[−19.180, −2.514]
Group	18.014	2.339	.591	.000	[13.367, 22.660]
DBC score	1.095	.193	.405	.000	[.712, 1.479]
Coping behaviour disengage	5.278	2.131	.128	.015	[1.043, 9.513]

In the next sequential multiple regression, group was entered at step one and challenging behaviours at step 2, The substance abuse coping strategy was entered at step three. Substance Abuse Coping Strategy did not explain additional variance in psychological distress and was not significant a significant predictor of psychological distress (*R*^*2*^ change = 0.000, *F*(1, 91) = 0.002, *p* = 0.968). The final model explains 79.1% of the variance in psychological distress in caregivers (Adjusted *R*^*2* =^ 0.791) and was significant *F*(3,94) = 119.891, *p* = 0.000.

In the thirdsequential multiple regression analysis, group and challenging behaviours were entered at step one and step two. In the third model, behavioural disengagement strategy was added explained additional over group and challenging behaviours and explained significant variance in parental psychological distress(*R*^*2*^ change = 0.013, *F*(1,89) = 6.131, *p* = 0.015). The final model explains 80.3% of the variance in psychological distress in caregivers (Adjusted *R*^*2*^ = 0.803) and was significant *F*(3,92) = 125.835, *p* = 0.000.

### Qualitative Analysis

Table 7 below outlines the themes and sub-themes that emerged from the responses provided by caregivers to the open-ended survey questions.

**Table 7:** Themes and subthemes

Theme	Sub-theme
1 – Confusing messages and guidance	1.1—Telling it like it is 1.2—We are living with the uncertainty and unknown
2 – Loss of Freedom	2.1—Boredom and frustration changes behavior
3 – Unsupported and forgotten	3.1—No relief and practical support 3.2—Psychosocial wellbeing and support

#### Theme 1. Confusing Messages and Guidance

Individuals with neurodevelopmental disabilities and their families found it challenging to understand the government guidance, social distancing rules and restrictions, finding them confusing:“Trying to stick to the rules while others are clearly not, and this is confusing information for my son and upsets him.”(Participant: 1). In particular, they experienced difficulty with understanding the details of media.

messages, the social distancing rules and public health safety measures. Confusion and frustration was exacerbated by the perception that others were not interpreting or following the rules to the same extent: experience of how hard it is to follow the rules, particularly when others are not:, “Government not making it go away and people on the telly ignoring social distancing when we tell him he has to do it.”(Participant 2).

Parents reported that interpreting the fine details of the social distancing rules was difficult when children applied them in a rigid way:. It had become a bit of an obsession: “He is obsessed with social distancing—trying to tell him that it’s okay for our household to cuddle.”(Participant 3).

This rigidity extended to expectations of others in the community, in relation to social distancing. Perceived breaches in the rules were the cause of negative emotional responses in children with intellectual disabilities: “Social distancing, that other people don’t realise she is scared when we have gone out and that they don’t understand her need for 3 + meters. She is very scared and angry at other people breaking the rules—like playing with a football.”(Participant: 4).

Sub-theme: Telling it like it is:

The cognitive functioning of children posed a challenge to family carers when trying to explain constructs such as how viruses spread, cause ill health and death and why steps to control the virus need to be wide-ranging and substantial::

“I can say ‘no school, we need to stay safe’ but what exactly does that mean if you have no concept of a virus, of time, of school not being there, or if it will ever be there again.”(Participant 5).

Carers therefore needed to be able to explain news updates in ways that were more understandable way for their children, by: “translating into accessible language the daily briefings which are being obsessed over” (Participant2). This extended to explaining the simplest concepts that had the potential to cause distress if not fully understood: “That it is okay, and he will not catch the virus by, e.g. just leaving the house for a walk.” (Participant 6).

Participants reported the supports that they wished they had been able to access. The materials included developmentally appropriate and accessible information with visual supports and social stories: “Yes—very, very simple social stories. We tend to get nonsense passed to us that is suitable only for kids with a fair bit of comprehension. It’s really unfair.” (Participant 5) And, “I really need a kind of “countdown’ a ‘calendar’ of reduced restrictions (‘museum is open, the library is open, school is open, the restaurant is open’. To kinda make a timeline, a logical timeline.” (Participant 5).

Sub Theme 1.2. We are living with the uncertainty and unknown.

Caregivers have experienced difficulties and distress with living with the ongoing ambiguity and the lack of clarity. This is uncertainty around both the present and the future as a result of COVID-19. The significant lifestyle changes and difficulties individuals and families have faced, including in their education provision, working from the home, day-to-day routine and loss of activities, and planned life events. Within this, there are anxieties about the future world, and the process of life getting back to normal. It encompasses the difficulties in accepting that much is unknown about COVID-19 therefore being able to provide concrete answers to their children is not possible. This is particularly difficult for children who prefer certainty and view the world in in black and white terms. One participant simply said the hardest thing was, “That there is no end date.”(Participant 7). Another stated: “Social distancing. They want to know when it will end, and they struggle to understand that nobody knows. I struggle to explain these to them.”(Participant 8). And the need for certainty was described by another participant: “Constant forward planning and needing to know and understand what will be happening in future days, especially when activities are limited. Confusing having all the family at home every day.” (Participant 1).

#### Theme 2. Loss of Freedom

Individuals and carers have experienced a loss of freedom in terms of not being able to do favourite things, activities—the loss of seeing friends and family, and for some individuals no longer being able to leave the house. Many of the respondents reported the negative impact of a loss of structure, routine and activities: “lack of routine, lack of going places, lack of activities he can do.”(Participant 5). Another also mentioned the most challenging change was: “The isolation and severe sudden change in routine.” The loss of freedom seemed very abstract to some children: “Where it has come from and why if he washes his hands and does not have symptoms can he not go swimming or bowling etc.”(Participant 9).

Carers also reported the emotional impact of restrictions that had an exacerbated impact due to the specific needs of their child – needs which not recognized within the general guidance: “I was frustrated with restrictions on food purchases, e.g. for restrictive diet I needed more than 3 of some items to get him fed for one week—I almost cried. In early weeks when shelves were empty of a preferred food. Also, we lost our weekly supermarket delivery slot—we are not in an at-risk group, but I find getting out to shops more challenging than most people due to my ASD son needing looked after.”(Participant 6).

Sub-theme: 2.1 Boredom and frustration changes behaviour.

Participants reported the loss of freedom led to boredom and frustration, and the significant negative changes in behaviour. Carers spoke about increased levels of challenging behaviour with a range of difficulties including physical aggression, emotional dysregulation (meltdowns), disturbed sleep patterns and low motivation and activity. One participant summarises behaviour changes: “Not going to sleep. Controlling time spent on electronic devices, getting him to leave the house, personal care, falling out with friends online.”(Participant10).

It was notable that respondents spoke about behaviour related to environment factors associated with their child’s neurodevelopmental disability: “Aggressive outbursts and high sensory requirements for which we do not have the equipment to deal with.” (Participant: 11).

It is worth noting that not every single family had experienced higher levels of challenging behavior; one participant noted quite the opposite, “He is actually calmer and more relaxed without external pressures and school.” (Participant 6).

#### Theme 3. Unsupported and Forgotten

Carers reported a sense of feeling unsupported and forgotten during the pandemic and lockdown, and highlighted a perceived absence of support from social, health and education services. There was a general feeling that they have been left on their own to meet complex educational, sensory-related, medical and social care needs: “a lack of support from specialist services. No check-ins.” (Participant 12). Carers expressed worries that changes in routine and behaviour were not sustainable or had potential negative implications for the future: “Lack of contact from school. We are very on our own, and she feels safe, but she is really scared to go out, and I fear she will refuse to go back to school, she is quite socially isolated there and is happy without the confusion of school social life, but this is no way to go on.”(Participant 4).

Sub-theme. 3.1. No relief and practical support.

Participants experienced a significant loss of practical support and have had a lack of respite or relief from looking after their children with some of the things they were struggling most with:” not getting a break from my child.” (Participant 12). With family carers speaking about a lack of practical support in education and other areas:” Lack of additional support needs (ASN) from school, no prioritisation based on ASN diagnosis, work can’t be done independently.” (Participant 13) In addition, “Losing all our face to face medical support that was frequent.”(Participant 7). Another participant noted: “Having to deal with the altered behaviour of our son with no respite or support from professionals or medical people.” (Partcipant 14).

Sub-theme. 3.2. Psychosocial well-being and support.

Respondents described the impact of not having their usual support networks on well-being both formal and informal due COVID: “Not having family to visit so I can have a different scene and someone to have a cuppa with” (Participant 15). There were some suggestions of supports that may have helped for family carers: “I’ve tried everything suggested! Maybe a peer supporter would have helped.” (Participant 16). And also, for the needs of their children, caregivers suggested: “someone out with the family to communicate with him through text or chat daily.”(Participant 17).

And, “More scaffolding (of worries), CBT for worries.”(Participant 12).

## Discussion

This study examined the effect of parenting a child with ID and the level of challenging child behaviours on parental psychological distress. The results align with those of similar studies, carried out before COVID-19, which indicate that caregiver psychological distress is positively associated with the severity of their child’s challenging behaviours (Blacher & McIntyre, 2006; Dunn et al., 2001; Gallagher & Hannigan 2014; Hastings et al., 2005; Powers, et al., 2002). The results also indicate that when group (ID or TD) and level of challenging behaviours are controlled for, parental coping strategies pose an additional risk for psychological distress.

Specifically, denial appears to be a maladaptive strategy relating to increased levels of psychological distress during the COVID pandemic, similar to previous research (Seltzer et al., 1995; Smith et al., 2008). This is in contrast to other studies which suggest that denial is potentially adaptive in the short term (Woodman & Hauser‐Cram, 2013) since it allows people to maintain well-being during stressful events beyond their control (Folkman & Lazarus, 1980). This impact on psychological distress may be due to denial (i.e. avoidance of the situation), being positively related to perceived parenting burden (Whittingham, et al., 2013). This indicates that when the caregivers are not acknowledging the reality of the stressful situation (i.e. COVID-19 pandemic and/or PHSM) (Carver, 1997; Carver et al., 1989) they perceive increased levels of burden, exasperating their stress levels.

In addition to denial, behavioural disengagement was also found to predict psychological distress in caregivers in the current study. This is similar to previous research which has found that this coping strategy increased levels of anxiety and depression in other groups of caregivers such as those who look after people with Alzheimer’s disease (García-Alberca, et al., 2012) and caregivers of children with epilepsy and cerebal palsy (Carona, et al., 2014). Although this coping strategy is rarely reported in caregivers of children with developmental disabilities (Woodman & Hauser-Cram, 2013) behavioural disengagement and helplessness may be reflective of individuals feeling trapped, or out of control (Usher et al., 2020), and, as the qualitative data suggests, being unsupported and forgotten.

The current study also found that both high levels of challenging behaviour by the child as well as behaviour disengagement by the caregiver, predicted higher levels of psychological distress, again similar to previous research in caregivers of children with autism (Lovell & Wetherell, 2015). This similarity may be explained by the high number of participants who were caregivers of autistic children that took part in the current study. This highlights the significant risks to the psychological well-being of family carers. It helps provide us with knowledge of how coping strategies and potentially increasing challenging behaviours during lockdown influences caregivers’ psychological distress. This is particularly important if the UK goes through another lockdown period as COVID-19 restrictions continue to ease.

In addition to the quantitative measures, qualitative answers provided by caregivers of children with neurodevelopmental disabilities in the current study showed that uncertainty around COVID-19 was difficult for the family to cope with and to communicate to their children (Koffman, et al., 2020). This uncertainty also extended to schooling and respite services, with caregivers unable to provide concrete dates to the children. The difficulties experienced may be associated with a rigid cognitive style and a lack of psychological flexibility, sometimes characteristic of individuals with autism (Fujino et al., 2019; Sethi et al., 2019). The study results corroborate and extend the finding that lack of respite and support has led to increased challenging behaviours in children and psychological distress in caregivers during the COVID-19 pandemic (Courtenay & Perera, 2020).

Confusing public health messages and guidance around the pandemic was a theme from the qualitative responses. Previous research carried out during an epidemic (i.e. the Severe Acute Respiratory – SARS epidemic in Taiwan) showed that increased levels of uncertainty and insecurity led to increased levels of anxiety in the general public (Ko et al., 2006). Many of the parents in the current study reported that their children with neurodevelopmental disabilities began fixating on virus risk and social distancing measures. This may be underpinned by the complex link between heightened anxiety and restrictive and repetitive behaviours (RRBs) in neurodevelopmental disabilities (Rodgers et al., 2012). RRBs refer to insistence on sameness, pre-occupation with a subject or object, and strict adherence to routines or rituals (APA, 2013) and have been proposed to play a role in the management of anxiety in neurodevelopmental disorders (Joosten, et al., 2009). Although RRBs can be observed in typically developing children (e.g. Çevikaslan et al., 2014) and children with various neurodevelopmental disabilities (e.g. Hepburn & MacLean, 2009) they are more often observed in autism (Knutsen, et al., 2019). Therefore, the reporting of this effect in the current sample may be reflective of the high proportion of caregivers of children with autism.

The lack of service provision, respite and school infrastructure as well as the presence of RRBs could also be related to some of the frustrations reported by caregivers, represented by the theme “loss of freedom”. The parents highlighted that not being able to do the child’s favourite activities or routines led to frustrations increasing challenging behaviours (Benson & Brooks, 2008; Minshawi et al., 2014; Tzischinsky et al., 2018). It should be noted, however, that some parents reported positive effects of the lockdown, with some parents reporting that more time at home had a calming and positive effect on their child, possibly due to reduced school-related anxiety (Cappadocia et al., 2012; Weiss et al., 2015). This would suggest that the impact of COVID-19 restrictions on children may be determined by the child’s existing characteristics and warrants further exploration to determine risk and protective factors in developing negative mental health outcomes.

Another key theme that emerged from the qualitative data included the lack of family, school, respite and medical support that happened during the COVID-19 pandemic. Relatedly, carers have often identified respite services as the most helpful service received and have been shown to prevent or lessen caregiver stress and burnout (Cowen, & Reed, 2002). The care is a source of relief since it offers care for a day, overnight, a few hours or several weeks at home or in an institution. It provides carers with an opportunity for a break, and a chance for them and other family members to revitalise themselves (Halpern, 1985). In addition to respite interventions, social support is also shown to mitigate negative effects of caregiver isolation and stress (Calvete & Arroyabe, 2012; Duis et al., 1997; Smith et al., 2012). Greater distress is reported in those families who perceive greater caregiver burden and limited social support (Dunn et al., 2001; Gallagher, et al., 2008). Therefore the reduced social support during the lockdown measures was particularly detrimental to caregiver’s mental health and indicates that if restrictive Lockdown was to occur again, other social and peer support must be put into place for this vulnerable group to reduce or mitigate these negative outcomes.

This study has several limitations. Firstly, the current study had a relatively low sample size due to the recruitment period within the most restrictive periods of lockdown in the UK (rather than the later transitioning phases). Despite this, several significant relationships and predictors following a conservative bonferroni correction were shown however caution should still be taken when interpreting these findings. There are also limitations when relying on parent-reported diagnoses; however, previous research has indicated that parent-reports are relatively reliable and robust (Rosenberg, et al., 2009). Thirdly there was a mixture of developmental diagnoses, meaning different child characteristics could have influenced the type and intensity of challenging behaviours that caregivers were exposed to (McClintock, et al., 2003) ultimately impacting their mental health outcomes. Therefore, future studies (collecting new data and using existing datasets) should try to examine whether the current findings extend to caregivers of all disability groups. Finally, given the nature of online survey methodology there are related limitations; participants could not ask for clarification so some questions may have been misunderstood. Also, because participants are self-selected, some responses may not be entirely representative of the UK.

Despite these shortcomings, the current study offers a crucial insight into the potential difficulties and barriers perceived by a vulnerable group such as caregivers of children with neurodevelopmental disabilities in the UK during COVID-19 restrictions. While levels of children’s challenging behaviours (i.e. indicators of children’s stress) continued to impact psychological distress in this vulnerable group, parental coping strategies were shown to further increase the risk of psychological distress As coping strategies are potentially modifiable, supporting caregivers in employing adaptive coping strategies has potential benefits for the transition out of covid-19 restrictions. Difficulties communicating aspects of the pandemic as well as the children’s adherence to rules and routine caused an increase in frustration and challenging behaviours in the children. This increase in challenging behaviours as well as lack of social, educational and professional support exasperated caregiver’s feelings of helplessness and denial during the pandemic leading to increased amounts of psychological distress. Lessons need to be learned so that if restrictions are re-introduced, these negative mental outcomes can be mitigated against and avoided by implementing appropriate support to protect the mental health of caregivers across the UK.
